# Proton and carbon ion radiotherapy for primary brain tumors delivered with active raster scanning at the Heidelberg Ion Therapy Center (HIT): early treatment results and study concepts

**DOI:** 10.1186/1748-717X-7-41

**Published:** 2012-03-21

**Authors:** Stefan Rieken, Daniel Habermehl, Thomas Haberer, Oliver Jaekel, Jürgen Debus, Stephanie E Combs

**Affiliations:** 1 Department of Radiation Oncology, University Hospital of Heidelberg, Im Neuenheimer Feld 400, 69120 Heidelberg, Germany; 2 Heavy Ion Therapy Center, University Hospital of Heidelberg, Im Neuenheimer Feld 450, 69120 Heidelberg, Germany; 3 Department of Radiation Oncology, Neuro-Radiation Oncology Research Group, University of Heidelberg, Im Neuenheimer Feld 400, 69120 Heidelberg, Germany

**Keywords:** Glioma, Meningioma, Particle therapy, Toxicity

## Abstract

**Background:**

Particle irradiation was established at the University of Heidelberg 2 years ago. To date, more than 400 patients have been treated including patients with primary brain tumors. In malignant glioma (WHO IV) patients, two clinical trials have been set up-one investigating the benefit of a carbon ion (18 GyE) vs. a proton boost (10 GyE) in addition to photon radiotherapy (50 Gy), the other one investigating reirradiation with escalating total dose schedules starting at 30 GyE. In atypical meningioma patients (WHO °II), a carbon ion boost of 18 GyE is applied to macroscopic tumor residues following previous photon irradiation with 50 Gy.

This study was set up in order to investigate toxicity and response after proton and carbon ion therapy for gliomas and meningiomas.

**Methods:**

33 patients with gliomas (n = 26) and meningiomas (n = 7) were treated with carbon ion (n = 26) and proton (n = 7) radiotherapy. In 22 patients, particle irradiation was combined with photon therapy. Temozolomide-based chemotherapy was combined with particle therapy in 17 patients with gliomas. Particle therapy as reirradiation was conducted in 7 patients. Target volume definition was based upon CT, MRI and PET imaging. Response was assessed by MRI examinations, and progression was diagnosed according to the Macdonald criteria. Toxicity was classified according to CTCAE v4.0.

**Results:**

Treatment was completed and tolerated well in all patients. Toxicity was moderate and included fatigue (24.2%), intermittent cranial nerve symptoms (6%) and single episodes of seizures (6%). At first and second follow-up examinations, mean maximum tumor diameters had slightly decreased from 29.7 mm to 27.1 mm and 24.9 mm respectively. Nine glioma patients suffered from tumor relapse, among these 5 with infield relapses, causing death in 8 patients. There was no progression in any meningioma patient.

**Conclusions:**

Particle radiotherapy is safe and feasible in patients with primary brain tumors. It is associated with little toxicity. A positive response of both gliomas and meningiomas, which is suggested in these preliminary data, must be evaluated in further clinical trials.

## Background

Despite continuously evolving extensive treatment concepts, primary brain tumors such as low grade and high grade gliomas, as well as meningiomas have not ceased to cause high morbidity and lethality due to biological aggressiveness or location in close proximity to critical structures. Radiotherapy is implemented in most glioma and many meningiomas therapy regimes and has been shown to significantly improve local control and prolong survival [1-3]. However, treatment results are still not satisfying, and most patients show tumor recurrence during the course of follow-up.

Particle irradiation is characterized by unique physical and biological properties which allow escalated dose deposition with steep gradients. Therefore, high local doses can be applied, while normal structures may be spared, and tumors in close vicinity of dose-limiting normal organs at risk may be treated more efficiently with higher doses. A clinical phase I/II trial from Japan has suggested high efficiency of carbon ion treatment for malignant gliomas with low toxicity [4]. For meningiomas, early data from our institution have demonstrated very promising results for atypical and anaplastic variants [5]. This has led to initiation of several trials that investigate a potential benefit of particle irradiation for both meningioma and gliomas [6-8].

The Heidelberg Ion Therapy Center (HIT) started patient treatment in November 2009 and has until now treated more than 250 patients with chordomas, chondrosarcomas, head and neck tumors, and primary brain tumors including both high and low grade gliomas and meningiomas. Carbon and proton irradiations have both been delivered as first radiotherapies but also as reirradiations [9,10]. Treatments so far were tolerated well with only moderate toxicity [11].

In the present manuscript we describe our currents institutional planning and treatment procedures in the management of particle irradiation for gliomas and meningiomas and analyse toxicity and early outcome of 33 patients treated within novel multimodal treatment concepts.

## Materials and methods

Patient characteristics are summarized in Table 1. Between November 2009 and January 2011, we treated 33 patients with gliomas and meningiomas at the HIT. Histological diagnosis was glioblastoma multiforme (GBM) in 18 (54.5%), anaplastic glioma in 3 (9.1%), low grade glioma in 5 (15.2%), and meningioma in 7 (21.2%) patients. Median age was 42 years (range 7-77 years) with 3 children ≤ 18 years included. Ten patients were female (30.3%), and 23 were male (69.7%). All patients provided written informed consent after thorough information about treatment concepts and possible side effects. For all study concepts, approval of the ethics committee of the University of Heidelberg had been obtained, as well as a positive votum by the Bundesamt für Strahlenschutz (BfS).

**Table 1:** 

patient characteristics			
		*[n]*	*[%]*
patient number		33	100
**gender**	female	10	30.3
	male	23	69.7
**age at RT**		*[years]*	
	median	42	
	range	7-77	
		*[n]*	*[%]*
pediatric patients [≤ 18 years]		3	9.1
**histology**	glioma	26	100
	WHO ° II	5	19.2
	WHO ° III	3	11.5
	WHO ° IV	18	69.2
	meningioma	7	100
	WHO ° I	3	43
	WHO ° II	3	43
	WHO ° III	1	14
**radiotherapy**	mixed modality	22	66.6
	[^12^C] *only*	6	18.2
	[^1^H] *only*	5	15.2
particle reirradiation		7	21.2
		*[ml]*	
mean particle volume [particle only, ml]		65.64	
mean particle volume [particle boost, ml]		69.29	
mean photon volume [particle boost, ml]		252.65	
		*[Gy]*	
range carbon total dose		18-45	
range proton total dose		10-57.2	
range photon total dose		50	
		[mm]	
Tumor diameter before RT		29.7	
Tumor diameter at first follow-up		27.1	
Tumor diameter at second follow-up		24.9	
		*[n]*	*[%]*
relapse meningioma		0	0
relapse glioma WHO ° II		0	0
relapse glioma WHO ° III/° III		9	42.3

Particle treatment for meningiomas was conducted in atypical meningioma patients (WHO °II) according to the MARCIE-protocol (7) following incomplete resection and was carried out as mixed modality irradiation with carbon ion boosts to the macroscopic tumor (6 × 3 GyE). For benign meningiomas, proton irradiation was offered in case of extensive cavernous sinus infiltration. In glioma patients with macroscopic tumor residues, carbon ion (6 × 3 GyE) vs. proton boosts (5 × 2 Gy) following previous photon radiotherapy (50 Gy) are being investigation within the CLEOPATRA trial (8). Reirradiation with carbon ions following a dose escalation schedule and starting a 10 × 3 GyE is offered to patients with benign and malignant unifocal glioma recurrences (WHO ° I-IV) (6).

Prior to treatment, individual fixation devices (Scotchcast-and Thermoplast-masks) were prepared to ensure precise daily positioning. Treatment planning was based on 3 mm CT slices fused with contrast agent-enhanced MRI. In case of gliomas, 18F-FET-PET/CT examinations were performed in order to support delineation of target volumes and to identify high-risk areas, whereas for meningiomas DOTATOC-PET/CT examinations were used to identify metabolically active tumor tissues by means of a lesion-versus-normal ratio (L/N ratio). Mean L/N ratio was 2.82.

For high-grade gliomas, a boost was defined with a gross tumor volume (GTV) including the contrast-enhacing lesion on MRI as well as the FET-PET-positive areas, adding a 0.5 cm safety margin for the clinical target volume (CTV_boost_). The CTV for photon radiotherapy (CTV_photons_) was defined as the T2-hyperintense areas adding 2-3 cm safety margins for microscopic spread.

For high risk meningioma, the GTV was defined as the contrast-enhancing areas on MR-imaging as well as the DOTATOC-positive areas, adding 5 mm margin for the CTV_boost_. The CTV_photons _was defined adding 2-3 cm to encompass potential microscopic spread.

The planning target volume (PTV) for particle therapy was calculated with a margin of 3 mm, for photon radiotherapy with a margin of 5 mm.

For particle boost irradiation, median boost volumes were 69.29 ccm, with additional photon volumes of 252.65 ccm. For sole particle treatment, the planning target volume encompassed a median volume of 65.64 ccm.

Seven patients were treated with protons, and 26 patients received carbon ion radiotherapy. Protons were chosen in case of children, low-grade meningioma and glioma and in one glioblastoma patient following randomisation (8). Single proton doses ranged from 1.8 to 2 GyE with total doses of 10-57.2 GyE. Single carbon ion doses were 3 GyE with total doses of 18-45 GyE. In case of either carbon ion or proton boost irradiation for either primary high grade gliomas [12] or high grade meningiomas [7], photon irradiation with 50 Gy was combined. In 7 patients, particle therapy was performed as reirradiation for recurrent tumors (2 × meningiomas, 5 × gliomas). Reirradiation for malignant gliomas was conducted according to a study protocol using carbon ions without combining with photon irradiation [6]. Two patients with recurrent meningiomas were treated with carbon ion total doses of 36 and 45 GyE. In 17 patients, particle treatment was combined with temozolomide chemotherapy at doses of 75 mg/m^2 ^body-surface-area 7 days per week during radiotherapy (1).

First clinical and MRI follow-up examination were performed six weeks after irradiation and every two months hereafter. Tumor response was assessed on the basis of T1-weighted MRI scans, and recurrence was diagnosed according to the Macdonald criteria. Acute toxicity arising during the first 90 days after radiotherapy completion was classified according to CTCAE v4.0.

## Results

### Workflow

**Figure 1:**
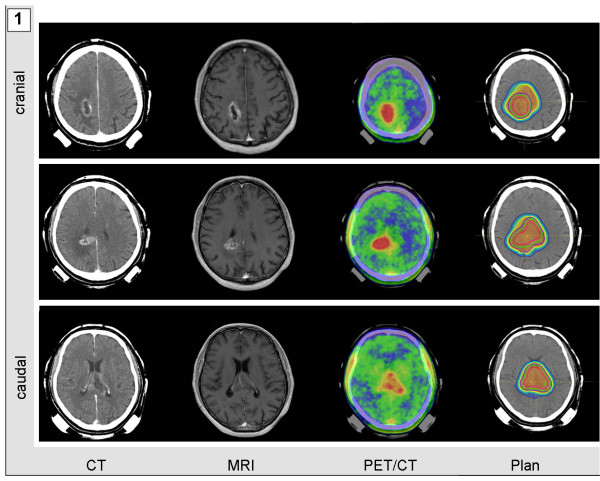
**Extensive glioblastoma multiforme in a 62-year-old man**. Contrast-agent enhanced CT and MRI scan were fused with a FET-PET/CT examination and used to calculate a two-beam carbon ion radiotherapy plan.

**Figure 2:**
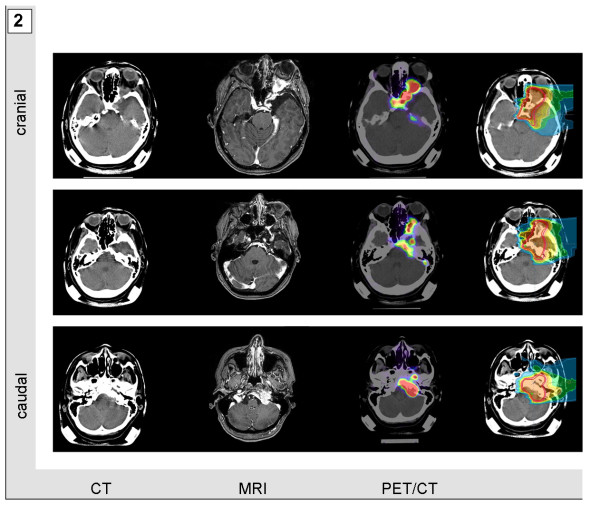
**Multifocal diffusely spreading atypical meningioma in a 55-year-old woman**. Contrast-agent enhanced CT and MRI scan were fused with a DOTATOC-PET/CT examination and used to calculate a two beam carbon ion radiotherapy plan.

**Figure 3:**
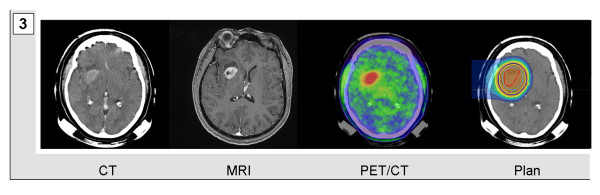
**Glioblastoma multiforme in the right frontal lobe of a 48-year-old woman**. Contrast-agent enhanced CT and MRI scan were fused with a FET-PET/CT examination and used to calculate a single beam carbon ion radiotherapy plan.

**Figure 4:**
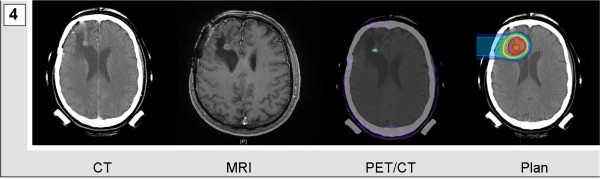
**Nodular atypical meningioma in a 50-year-old man**. Contrast-agent enhanced CT and MRI scan were fused with a DOTATOC-PET/CT examination and used to calculate a single beam carbon ion radiotherapy plan.

Mask fixations and planning examinations were completed about 2 weeks prior to the beginning of treatment. CT scans were performed without contrast agent to prevent miscalculation of particle range. For both morphological and functional identification of vital tumor tissue, gadolinium-enhanced MRI and PET/CTs-tracing either amino acid transporters in gliomas or somatostatin receptors in meningiomas-were performed and fused to the planning CTs. Target volumes included any contrast agent-enhanced structure in T1-weighted MRI-examination and were adapted to additionally include any region of increased PET tracer uptake. PET-associated target volume modifications caused expansion of target volumes in most cases of extensive tumor formations (Figure 1 and 2). Median volumes for particle treatment ranged from 17.16 to 434.98 ml with median volumes of 65.64 ml in case of sole particle treatment and 60.29 ml, when particle treatment was combined with photon radiotherapy. Tumor size itself was not a determining factor when allocating patients to particle treatment. However multifocality or systemic metastases for example via CSF dissemination were considered as contraindications for particle irradiation which in general was applied when local relapse was deemed the most likely threat to our patients. Figure 1, 2, 3, and 4 demonstrate four patients with malignant gliomas (Figures 3 and 1) and atypical meningiomas (Figures 4 and 2) of limited (Figures 3 and 4) and widespread (Figures 1 and 2) extension. Planning CT examinations (Figure 1, 2, 3, and 4; left) are fused with contrast-enhanced MRI and PET examination (Figure 1, 2, 3, and 4; central left and right) to generate particle irradiation treatment plans (Figure 1, 2, 3, and 4; right).

### Toxicity

Treatment was performed without interruptions in any patient. Acute toxicity was moderate and comprised low grade edema-related headache (14.7%) and increased tiredness during the day (24.2%). In two patients, unverified intensifications of pre-existing cranial nerve palsies were reported (1 × reduced visual acuity, 1 × reduced acoustic acuity). Visual impairment quickly recovered after oral administration of corticosteroids. Two patients suffered from single and self-limiting seizures during treatment. Apart from one temozolomide-related thrombocytopenia < 20,000/nl, no toxicities exceeding CTCAE v4.0 grade II were observed. The addition of chemotherapy was tolerated very well and did not enhance treatment toxicity.

### Response

**Figure 5:**
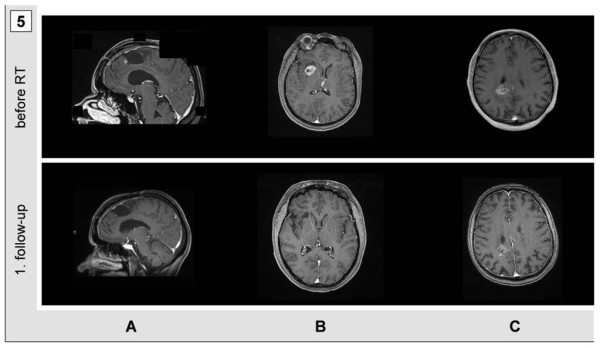
**Tumor response at 12 weeks after particle therapy in 3 individual glioblastoma patients**. A: reirradiation of a right frontal glioblastoma relapse with 10 × 3 GyE. B: combined photon/proton radiotherapy (total dose 60 GyE) with a proton boost irradiation with 5 × 2 GyE. C: combined photon/carbon ion radiotherapy (total dose 68 GyE) with a carbon ion boost irradiation with 6 × 3 GyE.

Median follow-up was 4.5 months. Early assessment of tumor response 6 and 12 weeks after radiotherapy demonstrated a slight, but not yet significant decrease in tumor diameters from 29.7 mm to 27.1 mm and 24.9 mm, respectively. Nine of eighteen glioblastoma patients (50%) suffered from progression of disease following particle radiotherapy, causing death in 8 patients (44.4%). Among these, 5 patients developed tumor recurrence within the particle radiotherapy fields (27.7%). There was no relapse in WHO °II/°III-glioma or meningioma patients during the present follow up. At the time of this analysis, neither age, sex, nor modality (^12^C vs. ^1^H) were significant indicators of response. Figure 5 demonstrates 3 individual glioblastoma patients who underwent different regimes of proton and carbon ion irradiation and who responded well to particle irradiation (Figure 5). After carbon ion RT either for reirradiation of recurrent glioblastoma (Figure 5A, 30 GyE) or for boost irradiation (Figure 5C, 18 GyE) following prior photon radiotherapy with 50 Gy photons, a significant decrease in tumor size, but also significantly reduced contrast agent uptake can be noted. Figure 5B shows a 47-year old female glioblastoma patient, who received a 10 GyE proton boost after irradiation with 50 Gy photons and who responded very well without suffering from treatment-related toxicities.

## Discussion

In the present manuscript we analysed daily workflow in planning and conduction of particle radiotherapy for brain tumors as well as toxicity and early response in 33 patients treated at the department of Heavy Ion Therapy (HIT) at the university hospital of Heidelberg.

The HIT started patient treatment in November 2009 and has treated more than 250 patients until today [9]. Treatment has been integrated into daily routine at the Department of Radiation Oncology and particle radiotherapy has been tolerated well with only moderate toxicity [11]. In primary CNS malignancies particle irradiation has proven beneficial outcome at low toxicity including malignant gliomas [4,13] and meningiomas [5]. Besides sole particle concepts, also combined particle-photon regimes have been established and have yielded promising results in gliomas [14] and meningiomas [15] in terms of toxicity, local control, and survival. At the HIT, several clinical trials have recently started accrual and will systematically analyse the impact of particle irradiation in both glioma and meningioma [6,7,12]. Before initiation of these studies, 33 patients have completed particle treatment and are being reported here.

Planning examination included functional biological imaging of tumor cell spread and viability by means of radiolabelled tracers. 18F-FET has been shown to possess a sensitivity of 94% in the diagnosis of malignant gliomas [16], despite its limited specificity that compares well to 18F-FDG [17]. Several authors have demonstrated a positive and prognostically relevant effect of considering amino acid uptake for target volume definition and have shown equivalence of both 11C-methionine and 18F-tyrosine in the evaluation of malignant gliomas [18-20]. Also, in menigioma patients functional imaging has been demonstrated to improve target volume delineation [21,22].

Carbon ion radiotherapy was offered to patients with high grade tumors. Carbon ion irradiation exerts very distinct radiobiological and radiophysical effects that translate into very precise dose deposition with increased biological effectiveness while simultaneously sparing closely neighbored organs at risk [23]. The beneficial effect of carbon ion radiotherapy in both malignant glioma and meningioma patients has been shown both preclinically [24,25] and in preliminary clinical trials [4,5]. Proton therapy was offered to children and patients with low-grade gliomas and meningiomas, where previous clinical trials have already demonstrated a beneficial impact [26-28].

Both carbon and proton treatments were tolerated well without differences regarding toxicity. For reason of modality selection, assigning low grade tumor patients to proton irradiation, these patients tended to fare better than patients treated with carbon ions.

As previously published by our group [11], acute sides effects were rare in brain tumor patients. Cranial nerve palsies as reported under particle irradiation [29] occurred temporarily in two patients. In one patient, reduction in visual acuity improved after administration of oral corticosteroids. In one further patient, who complained about hearing loss, no vestibulocochlear misfunction could be verified. General symptoms of CNS irradiation such as tiredness, reduced consciousness and dizziness occurred in 24.2%. Altered cognitive function affecting activities of daily life was reported in four patients (12.1%), all of whom were diagnosed with malignant glioma.

Only little time since treatment completion has elapsed and too little patient numbers have been included to provide reliable information about tumor response following carbon ion or proton RT. At this point, a slight but not yet significant decrease in median tumor size was observed. Nine patients suffered from tumor relapse following particle irradiation, including 5 (15.2%) with tumor recurrences within the particle irradiation fields indicating high tumor cell intrinsic radioresistance. Diagnosis was glioblastoma in all of them, and 3 patients (60%) had been treated with prior photon therapy and were patients for particle reirradiation. We failed to identify predictors of response in this heterogeneous patient group. However, even within this little cohort and not a surprise to the audience, it became clear that meningioma patients are characterised by higher progression free survival rates and thus a better prognosis than glioma patients. Miyatake et al. investigated toxicity and response of 6 patients with recurrent malignant meningiomas undergoing reirradiation by means of boron neutron capture therapy, which also represent a high LET therapy, and found radiological response in all patients after a time period of 7 to 13 months [30]. This strengthens the concept of high LET radiotherapies in patients with malignant meningiomas.

Further prognostically relevant factors remain to be identified in future clinical studies. In addition, longer follow-up periods are mandatory to evaluate normal tissue function after particle treatment and to solidify individual patterns of response when comparing proton and carbon ion treatments.

## Conclusion

Particle irradiation for primary brain tumors is safe and well tolerable. For adequate target volume delineation, multimodality imaging is helpful. Tumor response must be addressed in further clinical trials.

## Abbreviations

GyE: Gray equivalent; HIT: Heavy Ion Treatment Center; LET: Linear energy transfer; RBE: Relative biological effectiveness; PET: Positron emission tomography; DOTATOC: DOTA(0)-Phe(1)-Tyr(3)-octreotide; FET: O-(2-[F-18]Fluoroethyl-L-tyrosine.

## Competing interests

This work was supported by the Medical Faculty of the University of Heidelberg. Funding was also provided by the Deutsche Forschungsgemeinschaft (DFG), Klinische Forschergruppe Schwerionentherapie (KFO 214).

## Authors' contributions

SR performed clinical analyses, assisted in patient treatment and wrote the manuscript. DH helped to analyze patient data and organized follow-up examinations. TH and OJ were responsible for physical and technical conception of treatment plans. JD approved treatment plans, supervised patient treatment and financed the study. SC approved treatment plans, supervised patient treatment and helped to finalize the manuscript. All authors read and approved the current manuscript.
